# Supplementary material for: Immunomodulatory Responses of Subcapsular Sinus Floor Lymphatic Endothelial Cells in Tumor-Draining Lymph Nodes
Source: Cancers (Basel). 2022 Jul 24;14(15):3602. doi: 10.3390/cancers14153602 (PMC9330828; doi:10.3390/cancers14153602)
Supplement: Supplementary file 1 [file cancers-14-03602-s001.zip › Supplementary/Supplementary-Figures.pdf]

Supplementary Figure S1

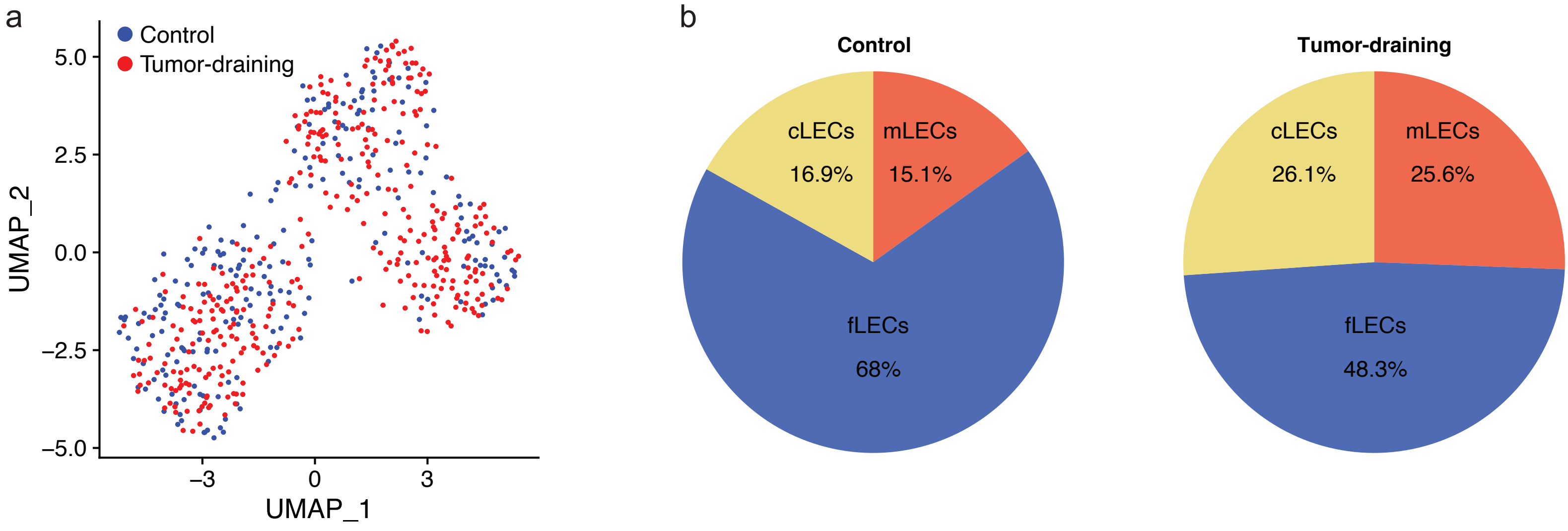

Supplementary Figure S1. ScRNA-seq of LECs from LNs draining tumor and healthy tissue. (**a**) UMAP visualization of LN LECs color-coded by condition, with 225 cells from control (blue) and 356 cells from tumor-draining (red) LNs. (**b**) Relative abundance of fLECs (blue), cLECs (yellow) and mLECs (red) in control and tumor-draining samples.

Supplementary Figure S2

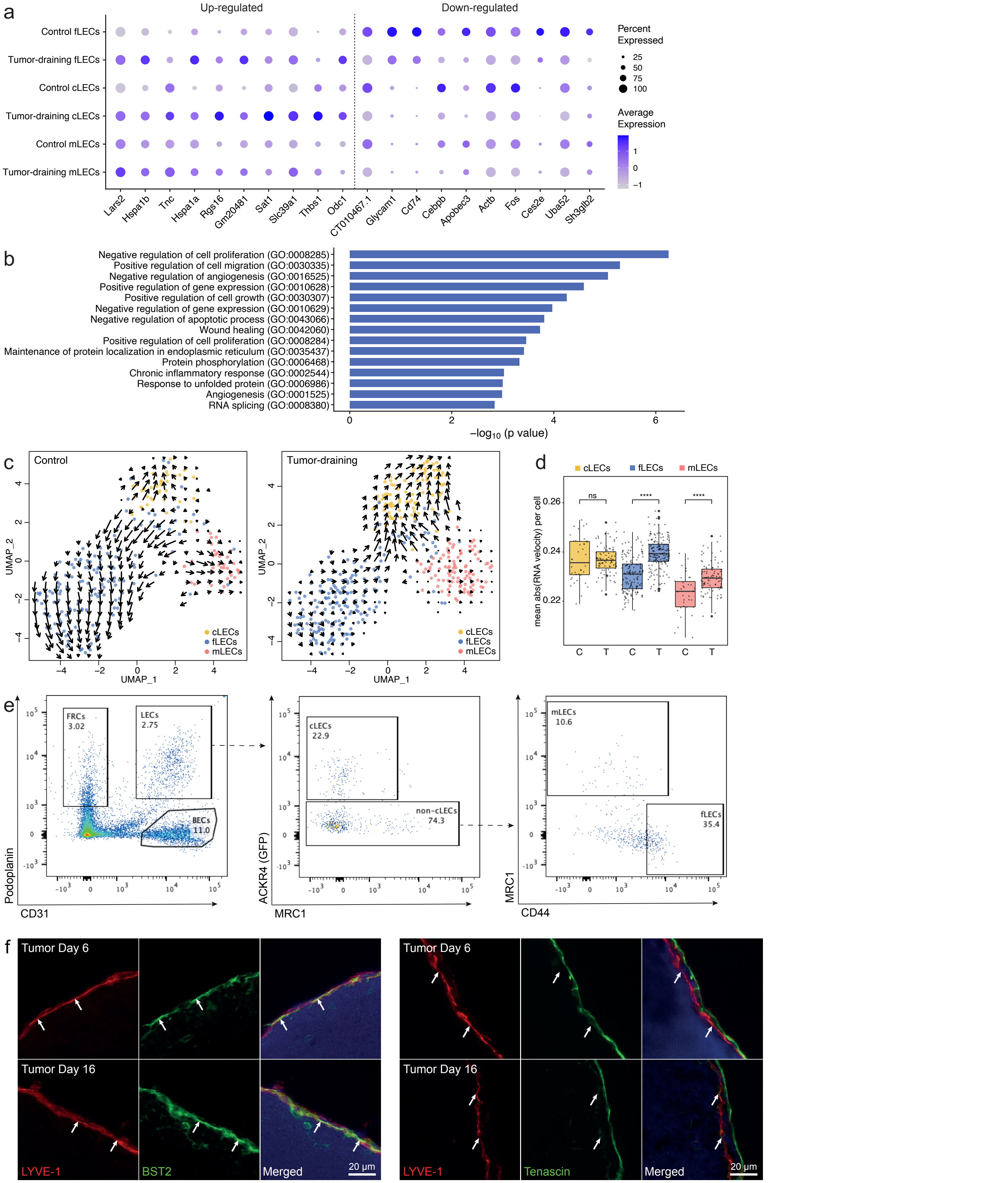

Supplementary Figure S2. GO analysis, RNA velocity and flow cytometry gating of fLECs, cLECs and mLECs. (**a**) Dotplot showing expression of the top 10 up- and downregulated genes in fLECs. (**b**) Top 15 gene ontology terms for biological processes enriched among genes upregulated in fLECs from tumor-draining compared to control LNs. (**c**) RNA trajectories of LECs from tumor-draining and control conditions projected onto UMAP. (**d**) Mean RNA velocity for each cell grouped by fLECs (blue), cLECs (yellow) and mLECs (red) from tumor-draining (T) and control conditions (C). (**e**) Gating strategy to differentiate LN stromal cell subsets FRCs, BECs, cLECs, fLECs and mLECs in LNs from Ackr4-GFP mice (pre-gated for CD45- living singlets). (**f**) Representative immunofluorescence stainings for LYVE-1 and BST2 or tensacin in tumor-draining LNs after 6 and 16 days. White arrows indicate the floor of the subcapsular sinus.

Supplementary Figure S3

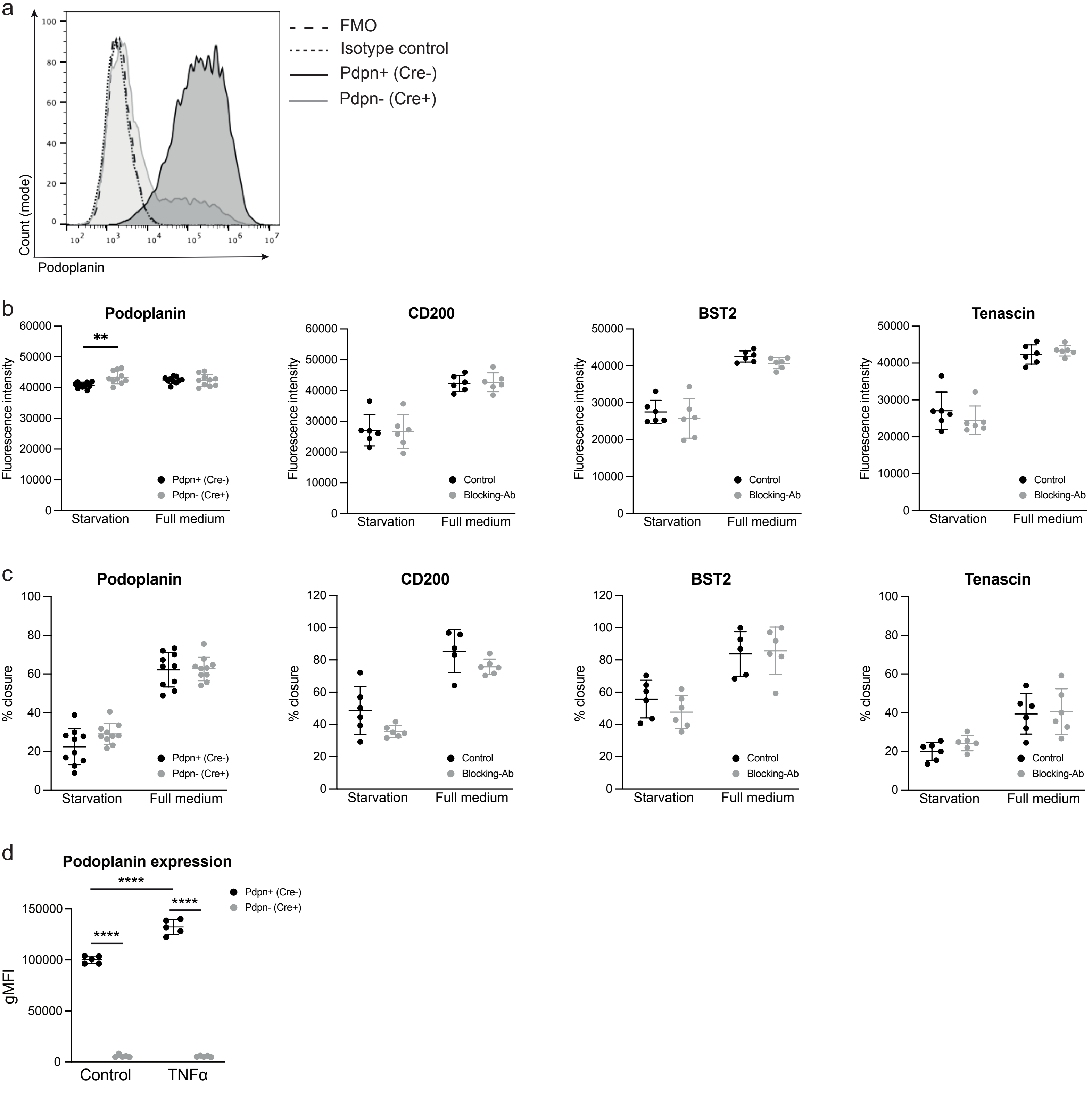

Supplementary Figure S3. Podoplanin deletion in LN LECs and *in vitro* viability and migration of LN LECs. (**a**) Podoplanin expression by LECs isolated from Cre- and Cre+ mice measured by FACS. (**b**) Viability of LN LECs with podoplanin deletion or upon antibody-mediated blockade of target genes (CD200, BST2 and tenascin). One representative experiment (of 3) is shown. Individual fluorescence intensity values (N = 6-10), their mean and SD are shown. (**c**) Migration of LN LECs with podoplanin deletion or upon antibody-mediated blockade of target genes measured by scratch assay. One representative experiment (of 3) is shown. Percentage of closed wound area per sample (N = 5-10), mean and SD are plotted. (**d**) Podoplanin expression by LECs isolated from Cre- (black) and Cre+ (grey) mice with and without stimulatiom with TNFα measured by flow cytometry. gMFI per sample (N = 5), mean and SD are shown. ** $p<0.01$, **** $p<0.0001$, unpaired t-test (b, c) or two-way ANOVA with Tukey's post-test (d).

Supplementary Figure S4

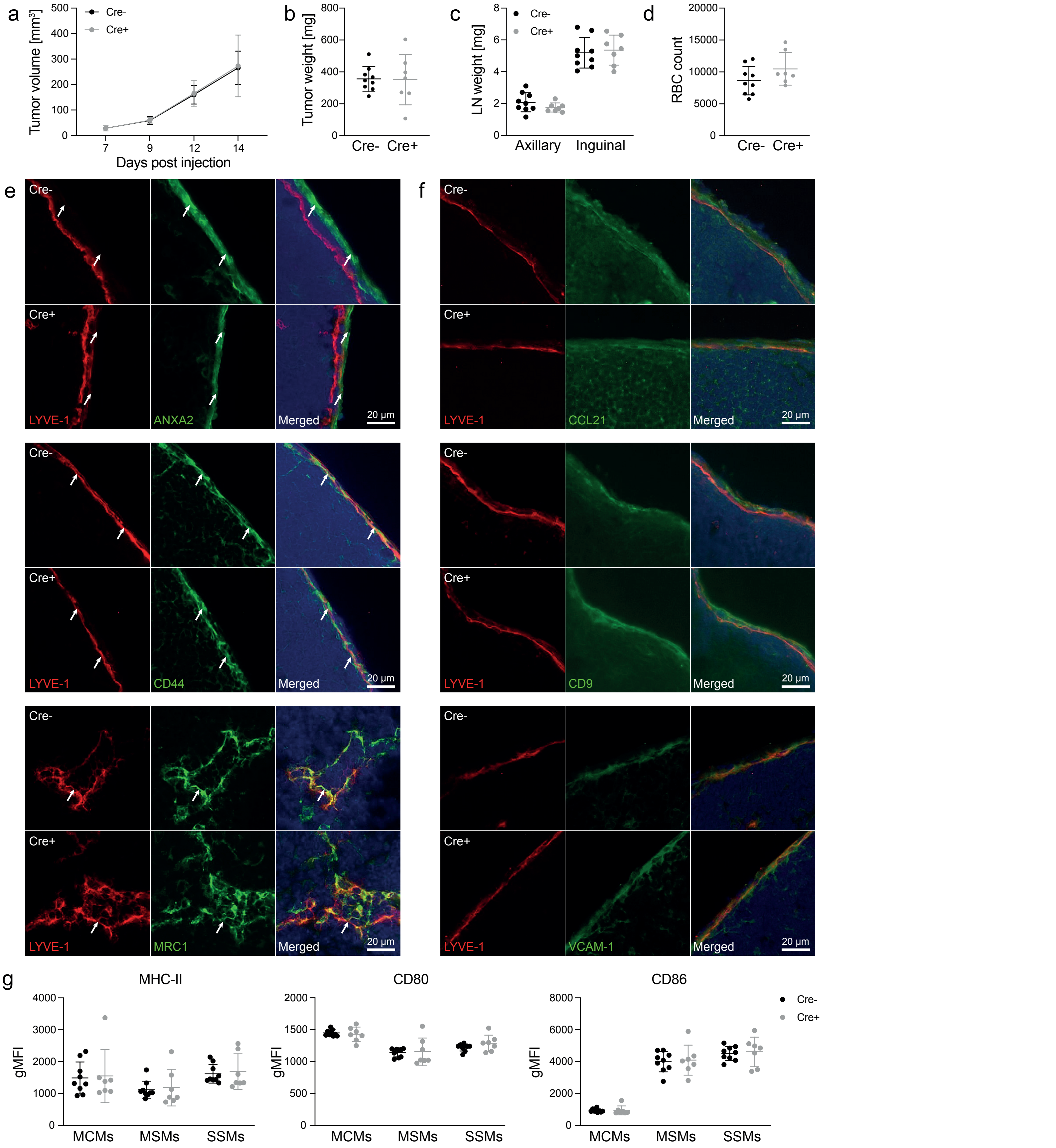

Supplementary Figure S4. Lymphatic podoplanin does not affect LEC identity, primary tumor growth and macrophage phenotypes. (**a-b**) Primary tumor volume (a) and weight on day 14 (b) in Pdpn$^{fl/fl}$ x Prox1-CreER^{T2} mice (Cre+). Pdpn$^{fl/fl}$ littermates (Cre-) served as controls (N = 10 Cre- / 8 Cre+). (**c**) Weight of tumor-draining LNs on day 14 (N = 9 Cre- / 7 Cre+). (**d**) Number of Ter119+ red blood cells (RBCs) in tumor-draining LNs determined by flow cytometry (N = 9 Cre- / 7 Cre+). (**e**) Representative immunofluorescence stainings for cLEC marker ANXA2, fLEC marker CD44 and mLEC marker MRC1 and LYVE-1 in tumor-draining LNs from Pdpn$^{fl/fl}$ x Prox1-CreER^{T2} mice (Cre+) and Pdpn$^{fl/fl}$ littermates (Cre-). White arrows indicate the respective LN LEC subset. (**f**) Representative immunofluorescence stainings of CCL21 and adhesion molecules CD9 and VCAM-1 in tumor-draining LNs from Pdpn$^{fl/fl}$ x Prox1-CreER^{T2} mice (Cre+) and Pdpn$^{fl/fl}$ littermates (Cre-). (**g**) Expression of MHC-II and co-stimulatory molecules CD80 and CD86 by LN macrophage subsets in tumor-draining LNs on day 14 (N = 9 Cre- / 7 Cre+).
